# Doxorubicin Induces a Senescent Phenotype in Murine and Human Astrocytes

**DOI:** 10.1111/jnc.70177

**Published:** 2025-08-05

**Authors:** Mariana Marques, Lívia de Sá Hayashide, Pedro Amorim, Beatriz Martins Fernandes, Ana Paula Bergamo Araujo, Daniel Fernandes Messor, Vitor Emanuel Leocadio, Bruna Pessoa, João Bastos Lima Pacca Corrêa, Cristopher Villablanca, René L. Vidal, Christian González‐Billault, Isadora Matias, Flávia Carvalho Alcantara Gomes, Luan Pereira Diniz

**Affiliations:** ^1^ Instituto de Ciências Biomédicas Universidade Federal do Rio de Janeiro Rio de Janeiro Brazil; ^2^ Cell and Neuronal Dynamics Laboratory, Department of Biology, Faculty of Sciences Universidad de Chile Santiago Chile; ^3^ Geroscience Center for Brain Health and Metabolism (GERO) Santiago Chile; ^4^ Center for Integrative Biology Universidad Mayor Santiago Chile; ^5^ The Buck Institute for Research on Aging Novato USA

**Keywords:** brain aging, human astrocytes, human brain and doxorubicin, senescence

## Abstract

Aging is a complex biological process that significantly impacts the central nervous system (CNS). Astrocytes, critical support cells in the brain, undergo senescence with age, contributing to neurodegenerative diseases. While previous studies have utilized murine models to investigate astrocyte senescence, human astrocytes offer a more physiologically relevant system to study age‐related neurodegenerative changes. This study presents a novel protocol for inducing senescence in both human primary and murine astrocytes using a combination of cellular stressors, such as lactacystin, H_2_O_2_, rotenone, and doxorubicin. Our results demonstrate that doxorubicin treatment effectively induces a robust senescent phenotype in both human and murine astrocytes, characterized by increased expression of senescence markers such as p21 and β‐galactosidase, along with activation of the DNA damage response (γ‐H2AX and 53BP1). Doxorubicin treatment increased nuclear size and induced cell cycle arrest in astrocytes, as revealed by reduced BrdU incorporation and decreased cell density, without inducing cytotoxic effects. This phenotype is accompanied by a pronounced pro‐inflammatory profile, with elevated expression of cytokines including MMP3, IL‐6, and IL‐1β, indicative of a strong senescence‐associated secretory phenotype (SASP). These findings provide a novel in vitro model for murine and human astrocyte senescence induced by doxorubicin, highlighting its relevance for studying mechanisms underlying age‐related neuroinflammation and neurodegeneration. By establishing a robust model of human astrocyte senescence, this study provides a valuable tool for exploring the molecular and cellular mechanisms driving age‐related neurodegenerative processes, serving as an alternative approach to traditional murine models.

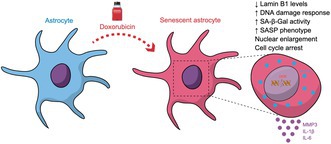

## Graphical abstract

Astrocyte senescence contributes to brain aging and neurodegeneration, yet human‐relevant models remain scarce. We developed a doxorubicin‐based protocol that reliably induces senescence in murine and human astrocytes. Treated cells showed nuclear enlargement, reduced proliferation (BrdU), and increased markers of senescence (p21, β‐galactosidase), DNA damage (γ‐H2AX, 53BP1), and inflammation (IL‐6, IL‐1β, MMP3), consistent with a senescence‐associated secretory phenotype (SASP). This model provides a robust tool to study astrocyte senescence and its role in age‐related neuroinflammation.

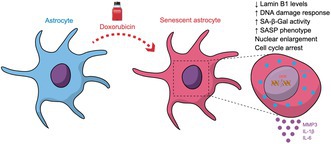

**Abbreviations**
53BP1: p53‐binding protein 1
AraC: cytosine arabinoside
ATP: adenosine triphosphate
DDR: DNA damage response
DIV: days in vitro
DMEM: Dulbecco's modified Eagle medium
DMEM/F12: Dulbecco's modified Eagle medium supplemented with F12 nutrient mixture
DRP1: dynamin‐related protein 1
FBS: fetal bovine serum
H_2_O_2_
: hydrogen peroxide
IL‐1β: interleukin‐1 beta
IL‐6: interleukin‐6
MMP3: metalloproteinase 3
PBS: phosphate‐buffered saline
ROS: reactive oxygen species
SASP: (senescence‐associated secretory phenotype), and γH2AX (phosphorylated H2AX)
SA‐β‐gal: senescence‐associated β‐galactosidase

## 1 Introduction

Human brain aging is a complex and multifaceted process characterized by a progressive decline in cognitive function and a reduction in neuronal connectivity. A central cellular mechanism in brain aging is cellular senescence, a state of stable cell cycle arrest triggered by various stressors, including DNA damage, oxidative stress, and oncogene activation. In the brain, senescent cells, particularly astrocytes, accumulate over time, contributing to tissue dysfunction and neuroinflammation (Verkerke et al. 2021). This accumulation plays a significant role in the pathogenesis of age‐related neurodegenerative disorders, such as Alzheimer's and Parkinson's disease.

Astrocytes are among the most abundant glial cells in the brain, essential for maintaining neuronal health, regulating synaptic function, and supporting the integrity of the blood–brain barrier (Diniz, Matias, Siqueira, et al. 2019). When astrocytes become dysfunctional or enter a senescent state, they contribute to the pathogenesis of various neurodegenerative diseases, including Alzheimer's disease and Parkinson's disease (Diniz, Matias, Araujo, et al. 2019; Matias et al. 2019; Ungerleider et al. 2021). Their altered function in these states disrupts neuronal support and exacerbates neuroinflammation, further advancing disease progression, highlighting the need to understand and address astrocyte senescence in neurodegenerative conditions (Matias et al. 2019).

Human astrocytes exhibit notable morphological and functional differences compared to those of rodents, which make them particularly relevant for studying neurodegenerative diseases and aging in humans. Morphologically, human astrocytes are larger, have a more complex structure, and contain a greater number of processes than rodent astrocytes, enabling them to cover a larger area and connect with a significantly higher number of synapses (Oberheim et al. 2009; Degl'Innocenti and Dell'Anno 2023).

Functionally, human astrocytes display enhanced capabilities in supporting synaptic activity, regulating neurotransmitter cycling, and maintaining the blood–brain barrier (Diniz et al. 2012; Verkhratsky et al. 2023). These distinctions are critical as they allow human astrocytes to exert a more refined control over neural networks and neuroinflammatory responses. Currently, there are no well‐established models for inducing senescence in human astrocytes, limiting our ability to accurately study age‐related cellular dysfunction in these critical CNS cells.

Given the critical roles astrocytes play in CNS homeostasis, the development of robust and reproducible models to induce and study cellular senescence specifically in primary human astrocytes is of great importance. Such models can provide valuable insights into the mechanisms by which senescent astrocytes contribute to neurodegenerative processes and offer platforms for testing senescence‐modulating therapies. Despite the established utility of certain chemical inducers of senescence in other cell types, the effectiveness of these agents in inducing a stable senescence phenotype in astrocytes remains underexplored and inconsistent.

In this study, we sought to evaluate the efficacy of different chemical agents, including rotenone, lactacystin, and hydrogen peroxide (H_2_O_2_), in inducing cellular senescence in primary murine astrocyte cultures. Our results demonstrated that these agents were inconsistent in replicating classical senescence‐associated markers, showing variable β‐galactosidase activity, DNA damage response (DDR markers such as phospho‐H2A.X and 53BP1), reduced lamin B1 levels, and increased p21 expression, suggesting that these conditions are suboptimal for achieving stable senescence in astrocytes. In contrast, doxorubicin, a well‐established DNA‐damaging agent, consistently induced a robust senescent phenotype, evidenced by significant changes in all classical senescence markers and the upregulation of senescence‐associated secretory phenotype (SASP) factors, including IL‐1β, IL‐6, and MMP3, in both murine and primary human astrocytes. These findings underscore the challenges of inducing stable senescence in astrocytes, especially in human astrocytes, and emphasize the need for further optimization of senescence induction protocols tailored specifically for these cells. Establishing reliable models of senescence in human astrocytes is essential to uncovering the role these glial cells play in neurodegenerative disease progression and for advancing targeted therapeutic strategies to mitigate the harmful effects of cellular senescence in the CNS. Such models have the potential to provide crucial insights into the mechanisms underlying age‐related neuroinflammation and neurodegeneration unique to the human brain.

## 2 Experimental Procedures

### 2.1 Animals

All animals were bred and housed in a temperature‐ (22 C ± 1 C) and humidity‐controlled (50% ± 10%) environment, in groups of two to four per cage, with ad libitum access to food and water. Animals used in this study were derived from the animal facilities of the Instituto de Ciências Biomédicas (ICB/UFRJ) and Faculdade de Farmácia (UFRJ). Considering that animals were shared across different experiments whenever possible, we estimate that approximately 90–110 neonatal mice were used in total for this study. Tissues from the same set of animals were often allocated to multiple experimental conditions to minimize animal usage, in compliance with the 3Rs principles (Replacement, Reduction, and Refinement). Animal handling and experimental procedures were approved by the Animal Use Ethics Committee of the Federal University of Rio de Janeiro (CEUA‐UFRJ), under protocols 119/23. All procedures were conducted in accordance with the Brazilian Guidelines on the Care and Use of Animals for Scientific and Teaching Purposes (DBCA). Experiments were designed to generate groups of equal or similar size, using randomization analysis. The work strongly adhered to the 3Rs principles, and all animal studies are reported in compliance with the ARRIVE guidelines.

### 2.2 Primary Murine Cortical Astrocyte Cultures

Primary cortical astrocyte cultures were established from newborn Swiss mice (postnatal days 0–3, P0–P3) following previously described protocols (Matias et al. 2022). Newborn mice were euthanized by rapid decapitation without prior anesthesia, in accordance with approved ethical protocols (CEUA‐UFRJ, protocol 119/23), and their brains were immediately dissected. This method was chosen to avoid potential pharmacological interference in subsequent cellular and molecular analyses. Each primary culture was prepared using a pool of brains from 2 to 3 animals per experiment. Briefly, the cerebral cortices were isolated, and the meninges were carefully removed. The dissected cortical tissues were mechanically dissociated and seeded into 25 cm^2^ culture flasks and 24‐well plates containing glass coverslips, both previously coated with poly‐L‐lysine (Sigma‐Aldrich, cat. P2636) to promote cell adhesion. Cells were maintained in Dulbecco's Modified Eagle Medium supplemented with F12 nutrient mixture (DMEM/F12; Thermo Fisher Scientific) and 10% fetal bovine serum (FBS; Thermo Fisher Scientific). Cultures were incubated at 37 C in a humidified atmosphere containing 5% CO_2_ and 95% air for approximately 7–10 days in vitro (DIV), allowing the cells to reach confluence. The culture medium was replaced every 2–3 days. To analyze senescence in the long‐term culture model, cells were maintained under low oxygen conditions at 37°C in a humidified incubator set to 5% CO_2_ and 3.5% O_2_. To obtain purified astrocyte cultures, the cells were extensively washed with PBS, including vigorous tapping of the flasks during each wash step. Culture medium was replaced every 2 days, and repeated washes were performed to remove microglial cells. This protocol consistently yields highly purified astrocyte cultures, with approximately 90% GFAP‐positive and 99% S100β‐positive cells, and < 3% F4/80‐positive microglial contamination.

### 2.3 Primary Human Astrocyte Cultures

Commercially available human astrocytes (Thermo Fisher, cat K1884, lot. Lot 2 405 051) were used in this study. The cells were cultured in DMEM/F12 medium supplemented with 10% FBS on substrates pre‐coated with poly‐L‐lysine. The cells were maintained in vitro at 37°C in a humidified incubator with 5% CO_2_ and 95% air until they reached confluence. The culture medium was replaced every 2–3 days. At passage 1, the cells were expanded and cryopreserved. Cells were used for experiments up to passage 7. Routine morphological characterization of the cultures was performed, including immunostaining for astrocyte markers such as GFAP, glutamine synthetase, GLT1, S100β, and aquaporin 4, to confirm the astrocytic identity and purity of the cultures. Our cell lines are not listed in the ICLAC register of misidentified or cross‐contaminated lines.

### 2.4 Astrocyte Cultures Treatments

Confluent primary cortical astrocyte cultures were divided into several experimental groups for treatment. In one set of experiments, the groups were: control (treated with 0.1% PBS for 24 h), H_2_O_2_ (150 μM for 2 h), lactacystin (1 μM for 24 h), and rotenone (1 μM for 24 h), all in serum‐containing medium. After the treatment period, the treatments were removed, fresh medium was added, and the cells were maintained for an additional 72 h in vitro before fixation. The concentrations of rotenone, lactacystin, and hydrogen peroxide used in our study were based on previously published literature reporting their use in models of oxidative stress, proteasome inhibition, or mitochondrial dysfunction in glial cells (Bitto et al. 2010; Simmnacher et al. 2020). In another experiment, the astrocytes were divided into two groups: control (treated with PBS) and doxorubicin (Libbs Farmacêutica, Embu das Artes, São Paulo, Brazil), where cells were treated with 250 nM of doxorubicin for 72 h in serum‐containing medium. After this treatment period, the treatments were removed, fresh medium was added, and the cells were cultured for an additional 4 DIV. The culture medium was changed every 2 days, and 24 h before collecting samples, the cells were maintained in serum‐free medium.

### 2.5 SA‐β‐Gal Staining for Senescence Detection

In this study, we employed the Senescence β‐Galactosidase Staining Kit (Cell Signaling, Cat. #9860) to assess senescence‐associated β‐galactosidase (SA‐β‐Gal) activity in vitro. Cells were rinsed with phosphate‐buffered saline (PBS), fixed with the provided fixative solution, and stained with X‐gal staining solution overnight at 37°C. Blue staining indicative of SA‐β‐Gal activity was observed under a microscope. Quantification of X‐Gal color intensity was performed using the Threshold Color plugin in Image J software (NIH) (Figueiredo et al. 2013). Alternatively, the number of cells positive for X‐gal blue precipitate was compared to the total cells per frame and expressed as the ratio of cells positive for SA‐β‐Gal activity.

### 2.6 Immunocytochemistry

Astrocyte cultures were fixed with 4% paraformaldehyde (PFA) in PBS at pH 7.4 for 15 min. To block non‐specific binding sites, the cultures were then incubated with a solution of 3% bovine serum albumin (BSA), 5% normal goat serum, and 0.2% Triton X‐100 in PBS for 1 h at room temperature. Following blocking, the cells were incubated overnight with primary antibodies: mouse anti‐p53 (1:100 dilution, Thermo Fisher Scientific, Cat. #MA5‐12571, RRID: AB_10986581); mouse anti‐phospho‐Histone H2AX (1:300, dilution; Millipore, Cat. JBW301, RRID: AB_309864); rabbit anti‐53BP1 (1:1000 dilution, Novus Biologicals, Cat. NB100‐304, RRID: AB_350221); mouse anti‐p21 (1:200 dilution, Santa Cruz Biotechnology, Cat. sc‐6246, RRID: AB_628073); rabbit anti‐Ki67 (1:200 dilution, Abcam, Cat. Cat. Ab15580, RRID: AB_443209) and rabbit anti‐Lamin B1 (1:1000 dilution, Abcam, Cat. Ab16048, RRID: AB_443298). After thorough washing with PBS, secondary antibodies (Alexa Fluor 546 or 488‐conjugated goat anti‐rabbit/mouse IgG; Invitrogen) were applied at appropriate dilutions (1:1000 Alexa Fluor 555 or 1:300 Alexa Fluor 488) for 2 h at room temperature. Finally, nuclei were counterstained with Hoechst 33342 (Thermo Fisher Scientific, Cat. 62249) and the cells were observed using a Leica SPE confocal microscope, a Nikon TE2000 microscope, or a Nexcope NIB‐620FL microscope.

### 2.7 Immunofluorescence Analysis

Densitometry for the immunocytochemistry images was performed using integrated density values generated with the ImageJ program (National Institutes of Health, USA, RRID:SCR_003070). Immunocytochemistry data were collected from at least 10 fields per coverslip. The integrated density value was divided by the number of cells in each field. To evaluate the DDR analysis, the number of puncta was assessed using the *Puncta Analyzer* plugin (ImageJ). The number of puncta in each field was normalized to the number of DAPI‐stained cells, resulting in the number of puncta per cell. Alternatively, the number of cells positive for γH2AX foci (defined as ≥ 2 puncta per nucleus) was quantified relative to the total number of cells per frame and expressed as the proportion of γH2AX‐positive cells.

Nuclear area and perimeter were quantified in Hoechst‐stained astrocyte cultures using ImageJ (Fiji). For each culture, five random fields were selected at 60× magnification to avoid sampling bias. Nuclear contours were measured using the “Analyze Particles” function (measurements set to area and perimeter), excluding objects outside a biologically plausible size range. For each field, the mean nuclear area and perimeter were computed, and these field means were averaged across the five fields to yield a single value per culture. To facilitate comparison between experimental groups, values were normalized to the mean of the control group, which was set to 100%. At least three independent cultures per group were analyzed.

### 2.8 Cell Viability Assessment

We determined cellular cytotoxicity by measuring the extracellular activity of lactate dehydrogenase (LDH), which is an indicator of cellular injury. The LDH activity assay was performed using 50 μL of culture medium following the manufacturer's instructions (CytoTox‐Glo Cytotoxicity Assay, Promega, Cat. G1780).

### 2.9 BrdU Incorporation Assay

Astrocytes were incubated with 10 μM BrdU (Sigma, Cat. No. 11647229001) for 24 h at 37 C. After incubation, cells were fixed with FixDenat solution and processed according to the manufacturer's instructions. BrdU incorporation was detected using a peroxidase‐conjugated anti‐BrdU antibody, followed by colorimetric development with TMB substrate.

### 2.10 Quantitative RT‐PCR (qPCR)

The RNA from cortical astrocytes was isolated and purified using the ReliaPrep RNA Cell Miniprep System (Promega Corporation, USA; Cat. Z6012). The RNA was quantified using a NanoDrop ND‐1000 spectrophotometer (Thermo Fisher Scientific, Waltham, MA, USA). For reverse transcription, 1–2 μg of total RNA was used with the GoScriptTM Reverse Transcriptase cDNA reverse transcription kit according to the manufacturer's instructions (Promega Corporation, an affiliate of Promega Biotecnologia do Brasil, Ltda). Primers were designed and synthesized by IDT‐DNA (San Diego, CA, USA). The specific forward and reverse oligonucleotides used were as follows: Murine p21: (F) TCGCTGTCTTGCACTCTGGTGT e (R) CCAATCTGCGCTTGGAGTGATAG. Murine Lamin B1: (F) CAACTGACCTCATCTGGAAGAAC e (R) TGAAGACTGTGCTTCTCTGAGC. Murine RPLP0: (F) CAGGTGTTTGACAACGGCAGCATT e (R) ACTCAGTCTCCACAGACAATGCCA. Murine MMP3: (F) CTGAAGGAGAGGCTGACATA e (R) GAGCAGCAACCAGGAATAG. Murine IL‐1β: (F) CAGGCAGGCAGTATCACTCA e (R) TAATGGGAACGTCACACACC. Murine IL‐6: (F) CACTCTGGAGGAGAAACGGAAGG e (R) GCAGGCATGAGGCAAACAGTC. Human 18S: (F) ACCCGTTGAACCCCATTCGTGA e (R) GCCTCACTAAACCATCCAATCGG. Human MMP3: (F) CACTCACAGACCTGACTCGGTT e (R) AAGCAGGATCACAGTTGGCTGG. Human IL‐1β: (F) CCACAGACCTTCCAGGAGAATG e (R) GTGCAGTTCAGTGATCGTACAGG. Human IL‐6: (F) AGACAGCCACTCACCTCTTCAG e (R) TTCTGCCAGTGCCTCTTTGCTG. Quantitative real‐time PCR was performed using the Fast SYBR Green Master Mix qPCR Master Mix (Applied Biosystems TM) with the following cycling conditions: 95°C for 20 s, followed by 40 cycles of 95°C for 1 s and 60°C for 20 s, using the Quant Studio 7 Flex System (Applied Biosystems TM). The relative expression levels of the genes were calculated using the 2^−ΔΔCT^ method (Livak and Schmittgen 2001).

### 2.11 Data and Statistical Analysis

Sample sizes were not predetermined statistically but were chosen based on precedent in prior comparable studies, which consistently yielded significant results (Diniz et al. 2024; Matias et al. 2022). Statistical analysis of quantitative data was performed using GraphPad Prism software version 8.0 (GraphPad Software, La Jolla, CA, USA, RRID:SCR_002798). A 95% confidence interval was applied, with *p*‐values < 0.05 considered statistically significant. Except for the control group, results are presented as mean ± SEM or mean ± SD. The Shapiro–Wilk test was applied to assess normal distribution. For samples that did not pass the normality test, non‐parametric tests were conducted, specifically the Mann–Whitney *U* test. In experiments with normal distribution, the Student's *t*‐test, one‐way ANOVA, and Tukey's post hoc test was used for statistical analysis. Detailed information on *p*‐values, sample sizes, and statistical tests used for each experiment is provided in the figure legends. No test for outliers was conducted.

## 3 Results

### 3.1 Pharmacological Stressors Fail to Induce the Astrocytic Senescence Phenotype

**FIGURE 1:**
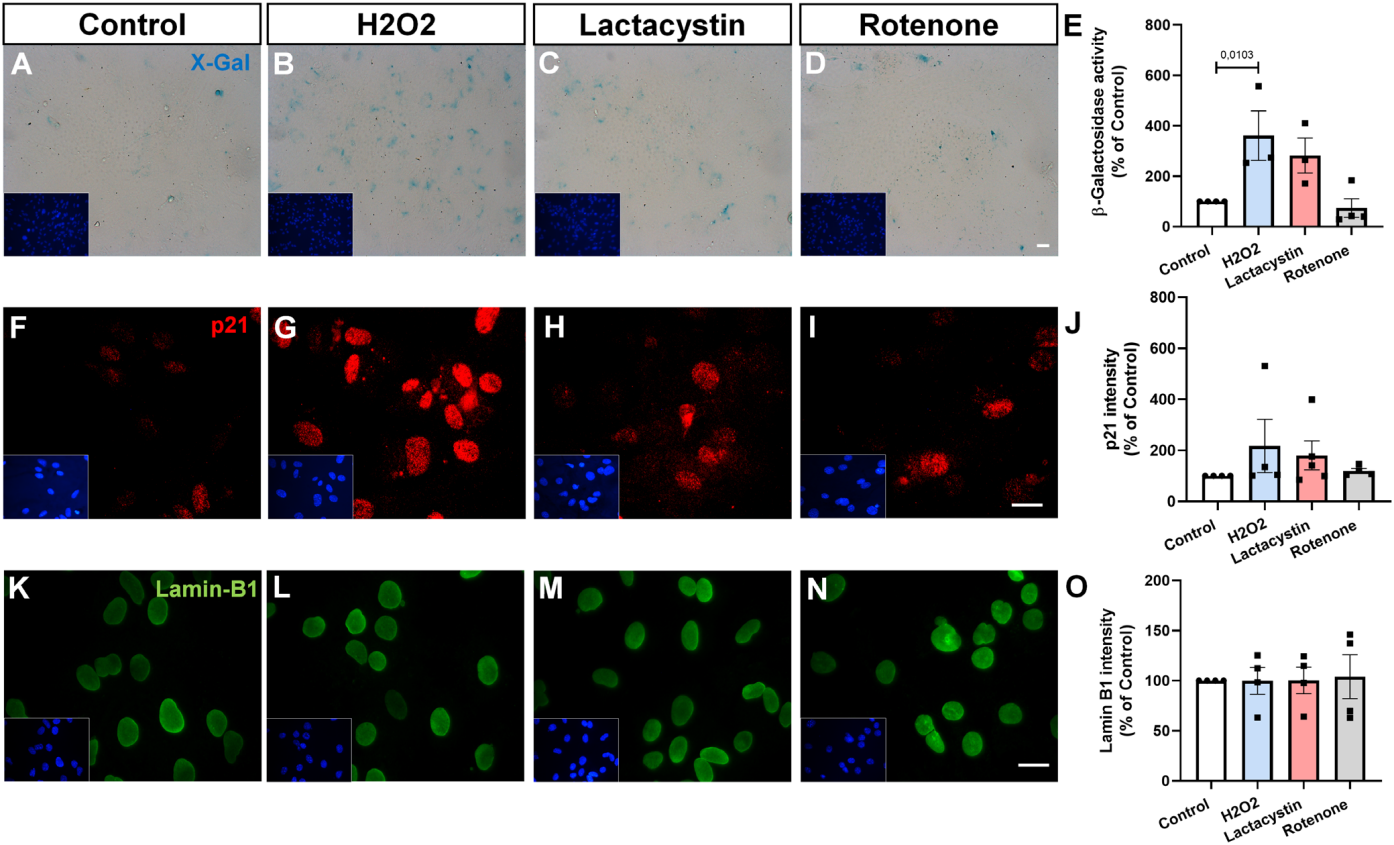
H_2_O_2_ induces increased β‐galactosidase activity in astrocyte cultures. Primary murine astrocyte cultures were treated with 150 μM H_2_O_2_ for 2 h, 1 μM lactacystin or rotenone for 24 h, or vehicle control (0.1% PBS, 24 h). (A–E) SAβgalactosidase assay reveals increased senescence‐associated activity in H_2_O_2_ treated cells. (C) Quantification: *F*(3, 10) = 6.494, *p* = 0.0103 (F–J) Immunocytochemistry for p21 shows no significant differences among groups (*F*(3, 13) = 0.7919, *p* = 0.5198; J). (K–O) Lamin B1 immunostaining indicates comparable expression across all conditions (*F*(3, 12) = 0.0188, *p* = 0.9963; O). Individual data points represent individual cultures (*n* = 3–5 cultures/group). Post hoc analysis was performed using Dunnett's multiple comparisons test. The *p*‐values shown in the graph refer to Dunnett's test versus control. Error bars indicate mean ± SEM. Scale bars, 20 μm.

Previously, our group demonstrated that astrocytes treated with cytosine arabinoside (AraC) and cultured for an extended period of 30 days in vitro exhibited a senescent phenotype (Diniz et al. 2024; Matias et al. 2022). Senescent cells are irreversible, non‐dividing cells characterized by morphological, biochemical, and secretory changes. Astrocytic senescence is characterized by several key cellular and molecular changes, including increased β‐galactosidase activity, commonly used as a senescence‐associated marker (SA‐β‐gal), and upregulation of cell cycle inhibitors such as p21, which plays a critical role in halting cell proliferation. A notable feature of senescent astrocytes is the loss of lamin B1, a nuclear envelope protein whose reduction is linked to nuclear instability. In this context, we tested the impact of pharmacological stressors previously described as being involved in cellular senescence phenotypes: hydrogen peroxide (H_2_O_2_), lactacystin (a proteasome inhibitor), and rotenone (a mitochondrial complex I inhibitor). In our current study, we observed that among the tested drugs, only H_2_O_2_ induced an increase in β‐galactosidase activity (Figure 1A–E), whereas lactacystin and rotenone had no effect on β‐galactosidase activity (Figure 1A–E). Moreover, none of the drugs had an effect on p21 levels (Figure 1F–J) or succeeded in reducing lamin B1 levels (Figure 1K–O).

**FIGURE 2:**
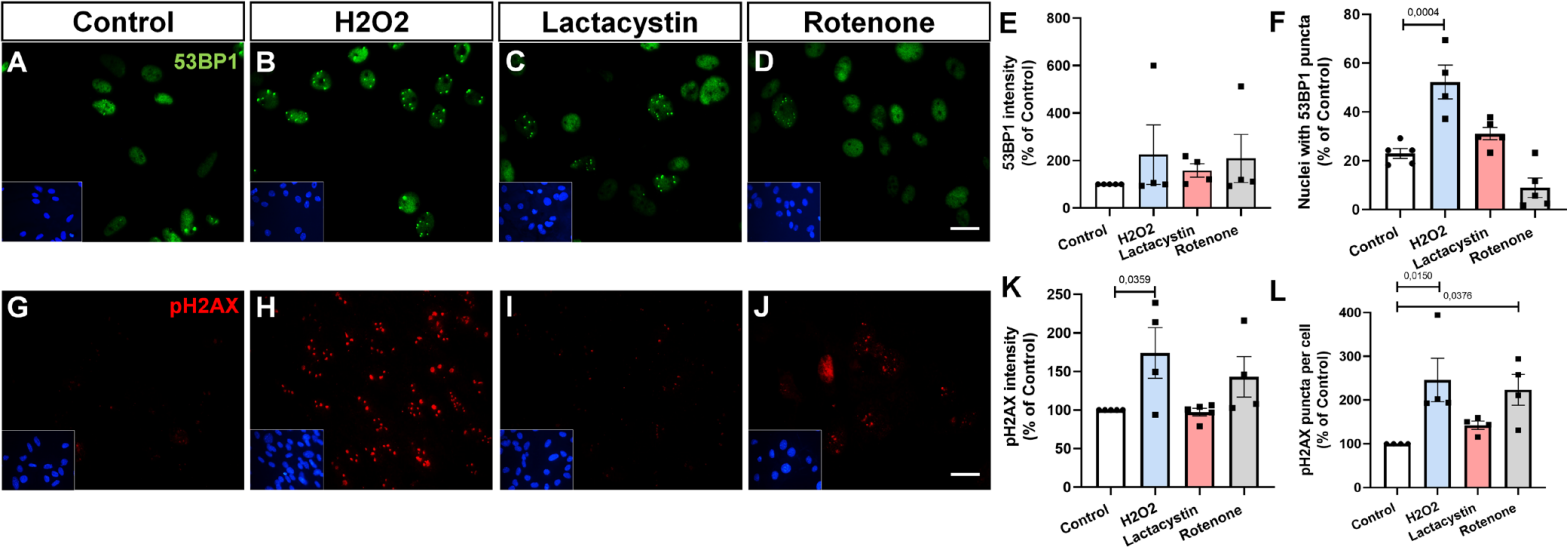
H_2_O_2_ and rotenone induce DNA damage response in astrocyte cultures. Primary murine astrocyte cultures were treated with 150 μM H_2_O_2_ for 2 h, 1 μM lactacystin or rotenone for 24 h, or vehicle control (0.1% PBS, 24 h). (A–F) Immunocytochemistry for 53BP1 shows similar overall intensity across experimental groups (*F*(3, 13) = 0.5825, *p* = 0.6369; E), although the number of nuclei with 53BP1 puncta is increased in the H_2_O_2_‐treated cultures (*F*(3, 15) = 19.78, *p* < 0.0001; F). (G–L) Immunostaining for γH2AX (pH2AX) reveals increased γH2AX intensity in H_2_O_2_‐treated cells (*F*(3, 14) = 3.872, *p* = 0.0330; K) and a significant increase in γH2AX puncta per cell in both H_2_O_2_ and rotenone treatments (*F*(3, 12) = 4.954, *p* = 0.0183; L). Individual data points represent individual cultures (*n* = 4–5 per group). One‐way ANOVA indicated a significant difference among groups. Post hoc analysis was performed using Dunnett's multiple comparisons test. The *p*‐values shown in the graph refer to Dunnett's test versus control. Error bars represent mean ± SEM; *p* < 0.05 versus control. Scale bars, 20 μm.

Furthermore, senescence leads to a DDR, characterized by increased levels of DNA damage markers such as 53BP1, a mediator of DNA repair, and phosphorylated H2AX (γH2AX), which indicates the presence of double‐strand DNA breaks. These changes collectively reflect the impaired functionality and heightened stress response typical of senescent astrocytes (Schneider et al. 2012). Within this context, we evaluated the impact of chemical stressors on the expression of 53BP1 and γH2AX in generating the DDR phenotype. For this purpose, the cells were treated with stressors, and the intensity and number of nuclear puncta were analyzed by immunocytochemistry. We observed that none of the stressors altered the overall intensity of 53BP1 staining (Figure 2A–E), however, we observed that only H_2_O_2_ treatment increased the number of 53BP1 nuclear puncta (Figure 2F), as well as both the intensity and number of γH2AX puncta (Figure 2K,L). In contrast, rotenone only increased the number of γH2AX puncta (Figure 2L), while lactacystin had no effect on the DDR (Figure 2A–L). In summary, these results indicate that the tested stressors were not effective in fully recapitulating the key features of the astrocytic senescence phenotype.

## 4 Doxorubicin Induces a Senescent Phenotype in Primary Astrocyte Cultures

**FIGURE 3:**
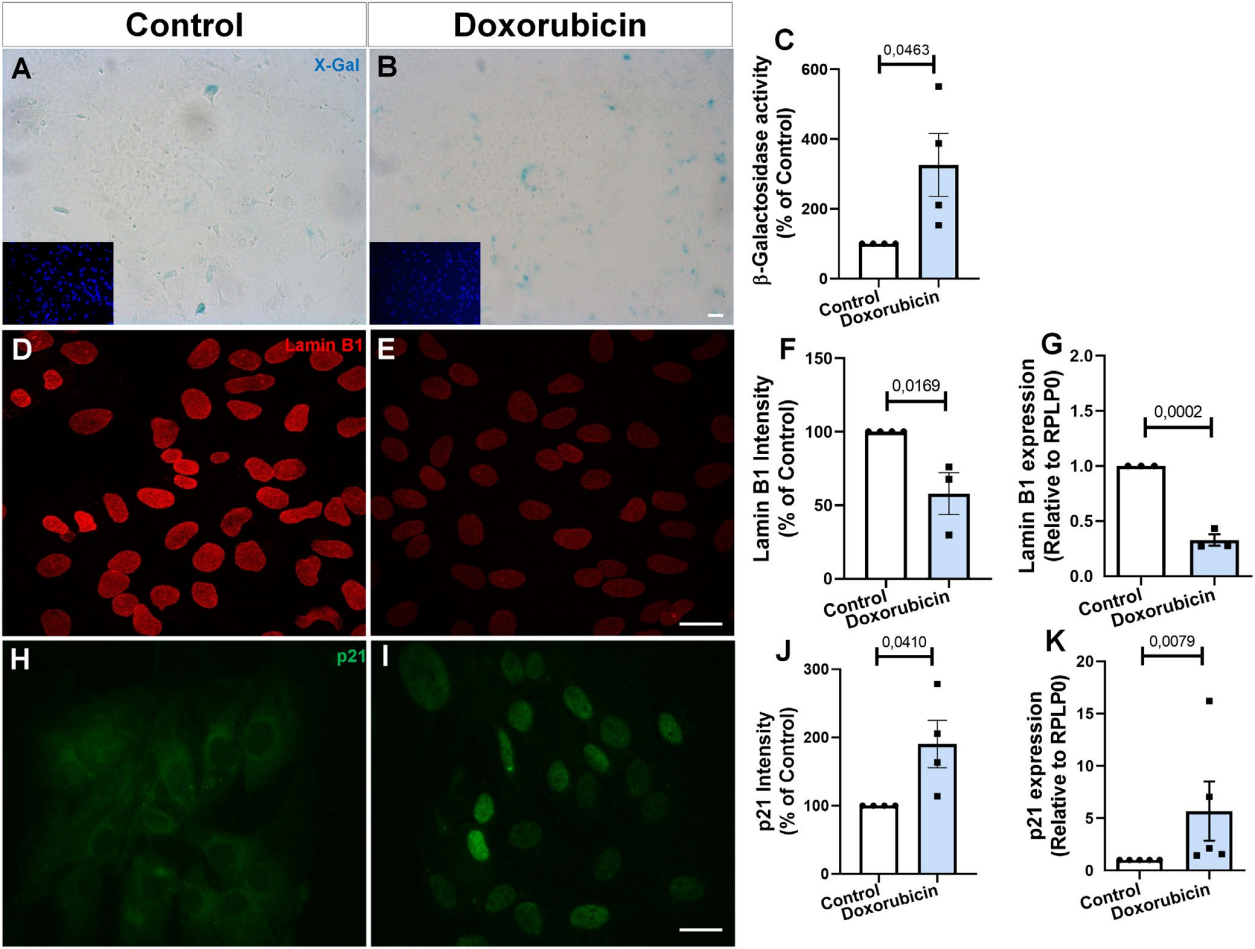
Doxorubicin induces a senescent phenotype in murine astrocytes. (A–C) Senescence‐associated β‐galactosidase (SA‐β‐gal) staining shows increased enzymatic activity in doxorubicin‐treated astrocytes compared to control. Quantification (C): T(6) = 2.504, *p* = 0.0463. (D–G) Lamin B1 levels were assessed by immunocytochemistry (D–F) and qPCR (G), revealing reduced signal intensity and transcript expression following doxorubicin exposure. (F): *t*(5) = 3.522, *p* = 0.0169; (G): *t*(4) = 12.79, *p* = 0.0002. (H–K) p21 levels increased in doxorubicin‐treated cultures, as shown by immunostaining (H–J) and qPCR (K). (J): *t*(6) = 2.594, *p* = 0.0410; (K): *U* = 0, *p* = 0.0079 (two‐tailed, exact). Bars represent mean ± SEM. Individual data points represent individual cultures (*n* = 3–6 per group). Statistical analysis was performed using unpaired two‐tailed Student's t‐test or Mann–Whitney *U* test (K). Scale bars, 20 μm.

Doxorubicin is a chemotherapeutic agent widely used in cancer treatment, known for its ability to intercalate into DNA and generate reactive oxygen species (ROS), leading to significant DNA damage. This damage can trigger a range of cellular responses, including apoptosis or, in some cases, senescence (Simmnacher et al. 2020) Doxorubicin is known to induce senescence in fibroblasts (Bientinesi et al. 2022). To investigate the ability of doxorubicin to induce senescence in astrocytes, we cultured primary astrocytes derived from the cerebral cortex for 7 days. Subsequently, the cells were treated with 250 nM doxorubicin for 72 h. Following treatment, fresh media was added, and the cells were cultured for an additional 4 days. The induction of a senescent phenotype by doxorubicin was confirmed by several key markers: elevated β‐galactosidase activity (Figure 3A–C), decreased lamin B1 staining (Figures 3D–F) and expression (Figure 3G), as well as increased p21 levels (Figure 3H–J) and expression (Figure 3K).

**FIGURE 4:**
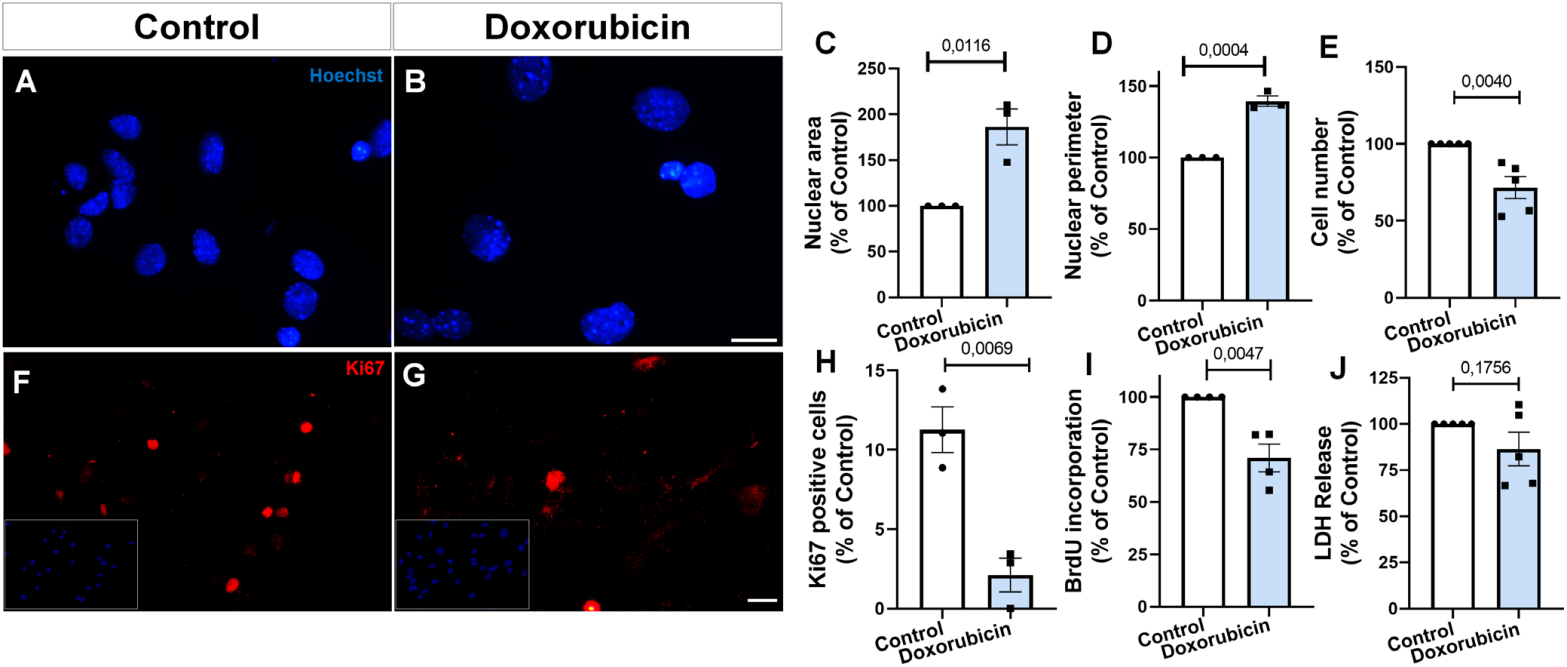
Doxorubicin induces nuclear enlargement and suppresses proliferation in murine astrocytes. (A, B) Hoechst staining shows nuclear morphology in control and doxorubicin‐treated cultures. (C, D) Quantification revealed increased nuclear area (*t*(4) = 4.410, *p* = 0.0116) and perimeter (*t*(4) = 10.95, *p* = 0.0004) after treatment. (E) Doxorubicin reduced total cell number (*t*(8) = 3.987, *p* = 0.0040). (F–H) Ki67 immunostaining shows decreased proliferation in treated cells. Quantification confirmed reduced percentage of Ki67+ cells (*t*(4) = 5.111, *p* = 0.0069). (I) BrdU incorporation was also decreased (*t*(6) = 4.372, *p* = 0.0047), indicating reduced DNA synthesis. (J) LDH release was not significantly altered (*t*(8) = 1.486, *p* = 0.1756), suggesting the absence of acute cytotoxicity. Bars represent mean ± SEM. Individual data points represent individual cultures (*n* = 3–5 per group) from independent cultures (*n* = 3–4 per group). Statistical comparisons were performed using unpaired two‐tailed Student's *t*‐test. Scale bars, 20 μm.

Cellular senescence is characterized by stable cell cycle arrest, nuclear enlargement, decreased proliferation, and the acquisition of distinct morphological and molecular features (Kumari and Jat 2021). Hoechst staining revealed that nuclei from doxorubicin‐treated cells were visibly enlarged compared to controls (Figure 4A,B). Quantitative analysis confirmed a significant increase in both nuclear area and perimeter relative to control (Figure 4C,D). Moreover, doxorubicin significantly reduced total cell number to approximately 70% of control levels (Figure 4E). Immunocytochemistry for the proliferation marker Ki67 showed a marked reduction in the number of Ki67‐positive cells in the doxorubicin group compared to control (Figure 4F–H). BrdU incorporation was also significantly decreased after doxorubicin exposure (Figure 4I), indicating impaired DNA synthesis and cell cycle arrest. In contrast, LDH release showed no significant difference between control and doxorubicin‐treated cells (Figure 4J), suggesting that doxorubicin did not induce acute cytotoxicity under these conditions.

To validate the cellular composition of our cultures, we performed immunocytochemical analysis for astrocytic and microglial markers. Nearly all cells in both control and doxorubicin‐treated conditions were positive for the astrocyte markers GFAP and S100β (Figure 1SA–D), indicating a highly enriched astrocyte population. In contrast, F4/80 immunoreactivity, a marker for microglia, was virtually absent in both groups (Figure S1E,F), suggesting minimal microglial contamination. Quantification confirmed that over 95% of cells expressed GFAP and S100β, while F4/80‐positive cells represented < 3% of the total population (Figure S1G). These data confirm that the observed effects of doxorubicin occurred in a predominantly astrocytic context.

**FIGURE 5:**
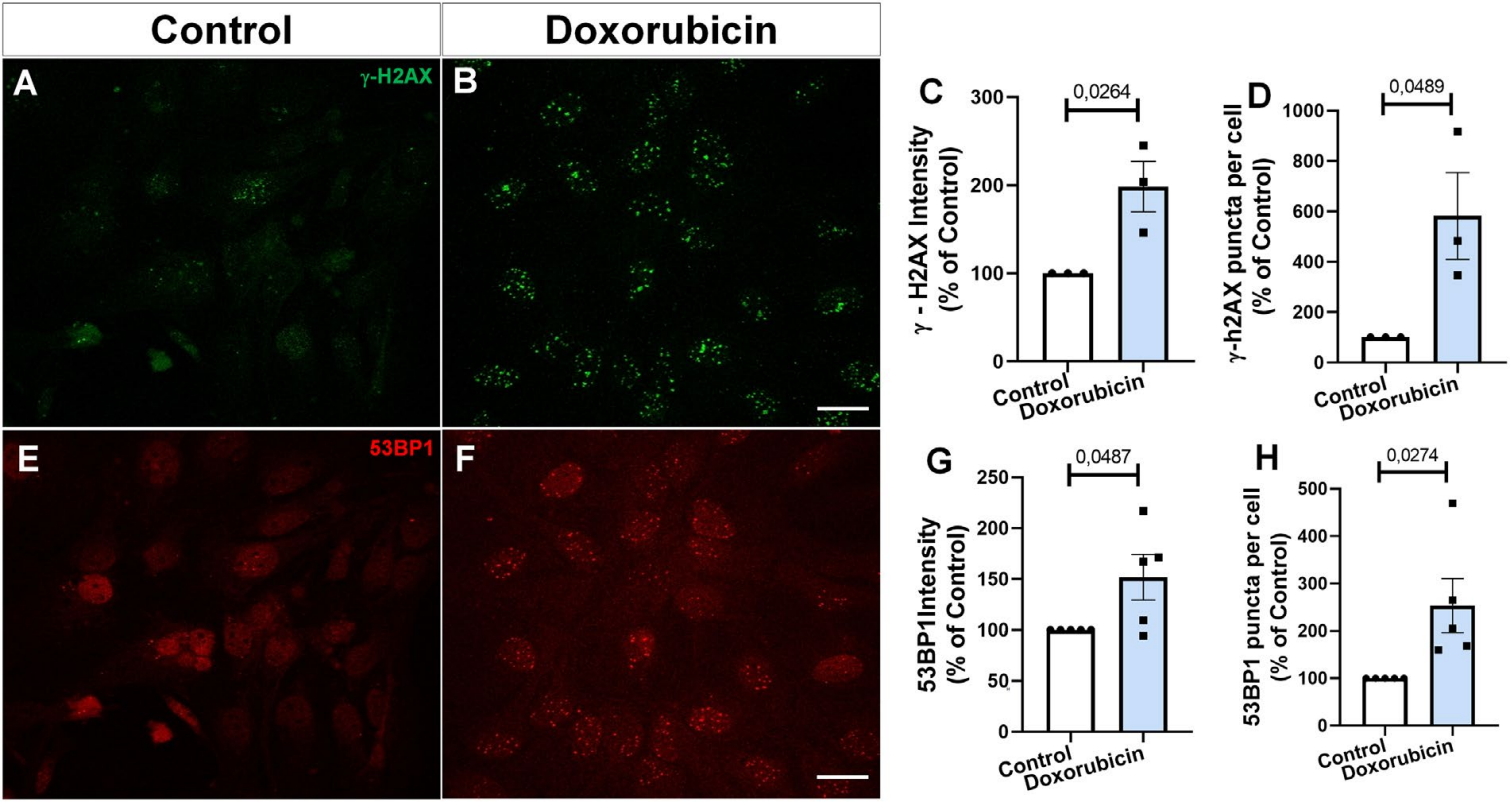
Doxorubicin increases DNA damage markers in murine astrocytes. (A–D) Immunostaining for γ‐H2AX revealed increased nuclear foci following doxorubicin treatment. Quantification confirmed elevated γ‐H2AX intensity (*t*(4) = 3.437, *p* = 0.0264; C) and increased number of puncta per nucleus *t*(4) = 2.799, *p* = 0.0489; D). (E–H) 53BP1 staining also showed enhanced DNA damage signaling in doxorubicin‐treated cells. Quantitative analysis indicated increased 53BP1 intensity (*t*(8) = 2.323, *p* = 0.0487; G) and number of 53BP1 puncta per cell (*t*(8) = 2.692, *p* = 0.0274; H). Bars represent mean ± SEM. Individual data points represent individual cultures (*n* = 3–5 per group). Statistical significance was assessed using unpaired two‐tailed Student's *t*‐test. Scale bars, 20 μm.

To assess the DDR in astrocyte cultures, we tested the effects of doxorubicin treatment by analyzing the expression of γH2AX and 53BP1 as markers of double‐strand DNA breaks. Confirming these findings, we observed that doxorubicin‐treated primary astrocytes exhibit activation of the DNA damage response, evidenced by increased staining intensity and distribution of nuclear puncta of γH2AX (Figure 5A–D) and 53BP1 (Figure 5E–H). Collectively, these results provide compelling evidence that doxorubicin treatment effectively induces a senescent phenotype in primary astrocyte cultures.

## 5 Doxorubicin Triggers the SASP Phenotype

**FIGURE 6:**
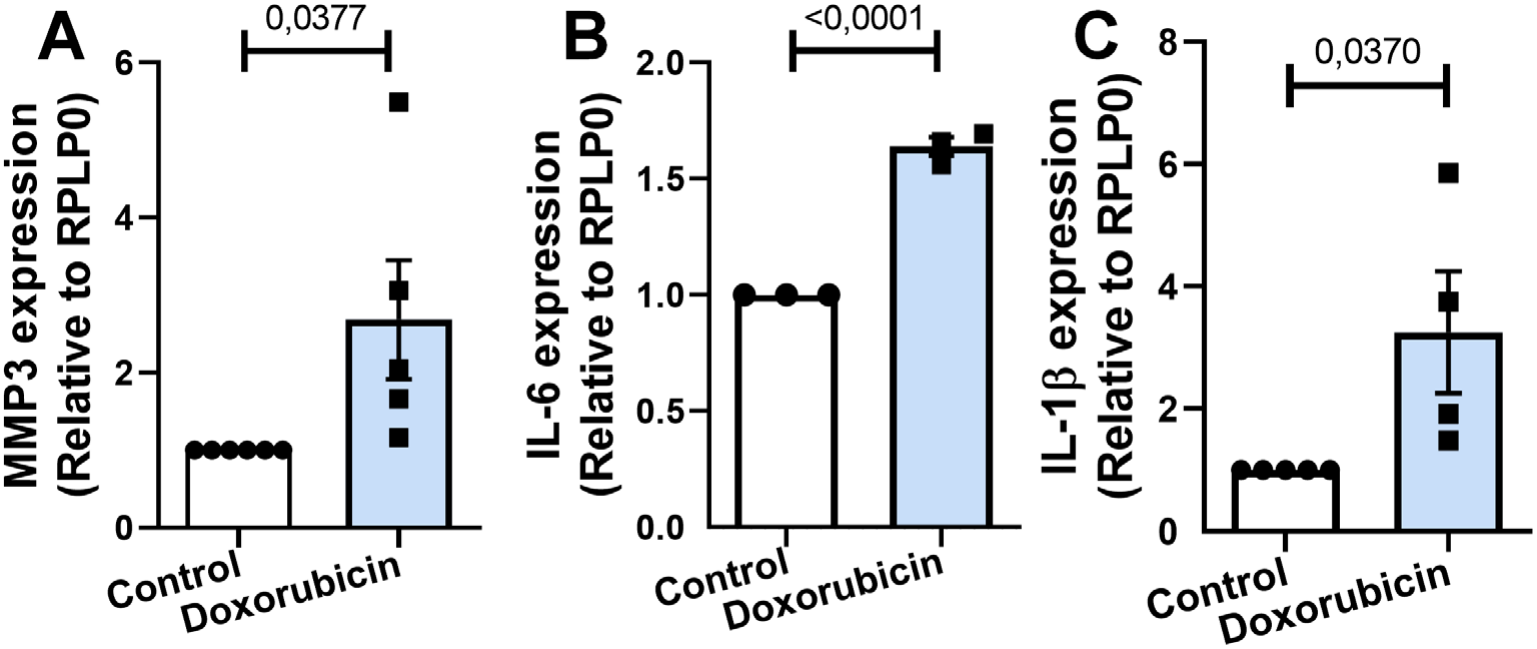
Doxorubicin induces SASP‐related gene expression in murine astrocytes. (A–C) qPCR analysis showed increased mRNA expression of SASP components following doxorubicin treatment. Expression of MMP3 (*t*(9) = 2.435, *p* = 0.0377; A), IL‐6 (*t*(4) = 15.94, *p* < 0.0001; B), and IL‐1β (*t*(7) = 2.570, *p* = 0.0370; C) was significantly elevated relative to control cultures. Bars represent mean ± SEM. Individual data points represent individual cultures (*n* = 3–6 per group). Statistical comparisons were performed using unpaired two‐tailed Student's *t*‐test. RPLP0: ribosomal protein lateral stalk subunit P0.

SASP is marked by the secretion of various pro‐inflammatory factors, including cytokines, chemokines, and matrix proteins. This secretory profile creates a microenvironment conducive to inflammation within surrounding tissues, triggering a cascade of systemic effects. SASP plays a critical role in the aging process (Ungerleider et al. 2021). As cells senesce, SASP contributes to the chronic, low‐grade inflammation characteristic of aging, often termed “senile inflammation.” In this study, we investigated whether doxorubicin treatment could induce the SASP phenotype in astrocyte cultures. Using qPCR analysis, we observed upregulation of Metalloproteinase 3 (MMP3) (Figure 6A), Interleukin 6 (IL‐6) (Figure 6B) and Interleukin 1 Beta (IL‐1β) (Figure 6C). These findings strongly suggest that doxorubicin treatment induces senescence and the associated SASP phenotype in astrocytes.

## 6 Doxorubicin‐Induced Senescence Is Maintained Long‐Term in Murine Primary Astrocytes

Senescent cells are known to exhibit resistance to various forms of programmed cell death, often resulting in their prolonged survival under in vitro conditions. To induce acute DNA damage, primary murine astrocytes were treated with 250 nM doxorubicin for 24 h, followed by a 14‐day recovery period under low‐oxygen conditions (5% CO_2_, 3.5% O_2_). This treatment led to pronounced senescence‐associated morphological changes, including cellular enlargement and increased lysosomal content, which correlated with a significant increase in the number of cells positive for senescence‐associated β‐galactosidase (SA‐β‐Gal) activity (Figure S2A–C). After 14 days, senescent astrocytes exhibited persistent DNA damage, evidenced by the presence of γH2AX foci in the majority of their enlarged nuclei (Figure S2D–F). Prolonged culture also resulted in reduced expression of Lamin B1 and elevated levels of phosphorylated p53, as determined by Western blot analysis (Figure S2G). Densitometric quantification using total protein (Stain‐Free signal) as a loading control confirmed a significant decrease in Lamin B1 levels alongside an increase in phospho‐p53 expression (Figure S2H–J).

## 7 Doxorubicin Induces Senescence in Human Astrocytes

Human astrocytes exhibit distinct morphological and functional differences compared to astrocytes from other species. Morphologically, human astrocytes are larger, have more complex branching, and form a more intricate network of interactions with neurons (Degl'Innocenti and Dell'Anno 2023). Studying senescence in human astrocytes is particularly important because it allows for a better understanding of how aging and cellular stress impact function in the human brain. To assess the effect of doxorubicin on the induction of a senescent phenotype, primary human astrocytes were cultured and treated with 250 nM of doxorubicin for 72 h. After the treatment, the media were refreshed, and the cells were cultured for an additional 4 days. Immunocytochemistry for GFAP confirmed that both control and doxorubicin‐treated cultures were composed predominantly of astrocytes, with approximately 95% of cells expressing GFAP, in agreement with the supplier's datasheet. Doxorubicin treatment did not alter the cellular identity of the cultures (Figure S3A–D), supporting that the effects observed upon doxorubicin exposure occurred in a predominantly astrocytic context.

**FIGURE 7:**
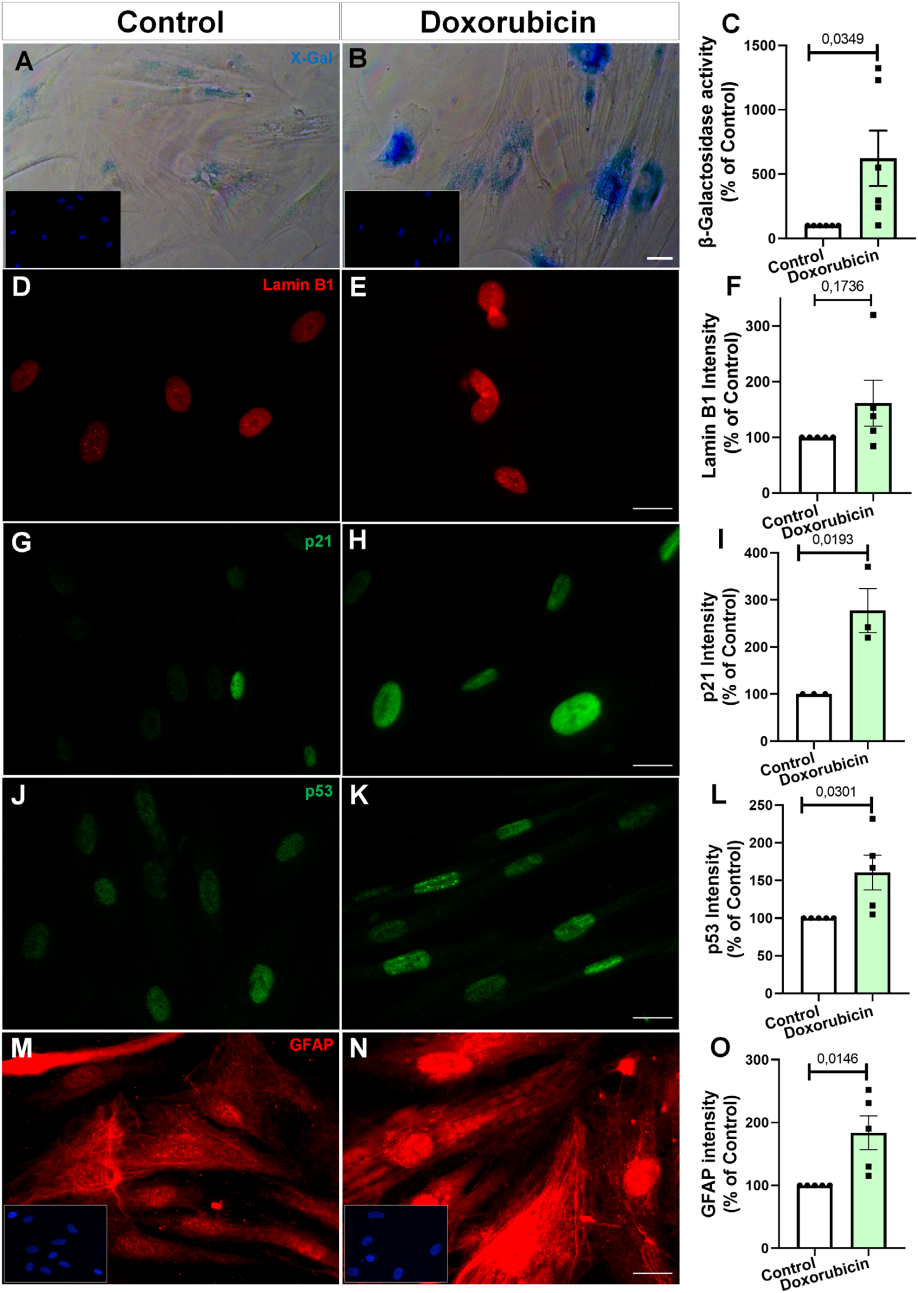
Doxorubicin induces senescence markers and GFAP upregulation in human astrocytes. (A–C) SA‐β‐galactosidase activity increased in doxorubicin‐treated astrocytes compared to control. Quantification (C): *t*(10) = 2.439, *p* = 0.0349. (D–F). Immunostaining for Lamin B1 revealed no significant difference between groups (*t*(8) = 1.494, *p* = 0.1736; F). (G–I) p21 immunoreactivity was elevated following treatment (*t*(4) = 3.790, *p* = 0.0193; I). (J–L) Doxorubicin also increased p53 intensity (*t*(8) = 2.632, *p* = 0.0301; L). (M–O) GFAP signal intensity was significantly higher in the treated group (*t*(8) = 3.104, *p* = 0.0146; O). Bars represent mean ± SEM. Individual data points represent individual cultures (*n* = 3–6 per group). Statistical comparisons were performed using unpaired two‐tailed Student's *t*‐test. Scale bars, 20 μm.

We observed that doxorubicin increased β‐galactosidase activity (Figure 7A–C), did not alter lamin B1 staining levels (Figure 7D–F), and led to elevated p21 levels (Figure 7G–I) as well as increased p53 level (Figure 7J–L). Immunocytochemical analysis revealed that doxorubicin treatment increased GFAP levels in human astrocytes, suggesting a reactive phenotype (Figure 7M–O).

**FIGURE 8:**
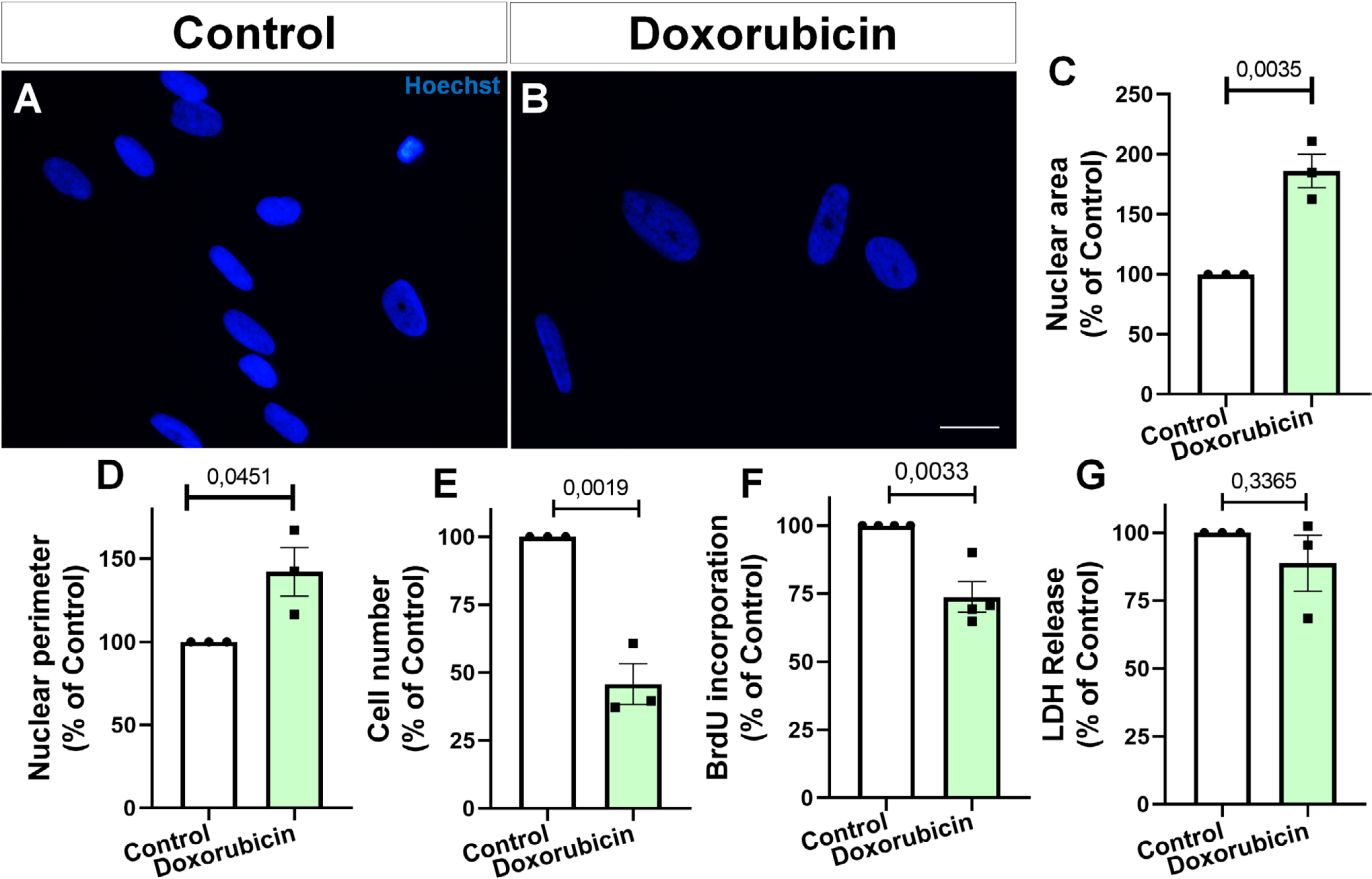
Nuclear morphology and cell proliferation are altered in human astrocytes following doxorubicin exposure. (A, B) Hoechst staining of nuclei revealed increased nuclear size in doxorubicin‐treated astrocytes. (C) Quantification showed increased nuclear area (*t*(4) = 6.170, *p* = 0.0035). (D) Nuclear perimeter was also elevated (*t*(4) = 2.878, *p* = 0.0451). (E) Total cell number was reduced in treated cells (*t*(4) = 7.249, *p* = 0.0019). (F) BrdU incorporation was significantly decreased, indicating reduced proliferation (*t*(6) = 4.697, *p* = 0.0033). (G) LDH release showed no significant difference (*t*(4) = 1.091, *p* = 0.3365), suggesting absence of overt cytotoxicity. Bars represent mean ± SEM. Individual data points represent individual cultures (*n* = 3–4 per group). Statistical analysis was performed using unpaired two‐tailed Student's *t*‐test. Scale bars, 20 μm.

The treatment of human astrocytes with doxorubicin resulted in a marked enlargement of nuclear morphology (Figure 8A,B), as evidenced by an approximately 1.9 fold increase in nuclear area (Figure 8C) and a 1.4 fold increase in nuclear perimeter relative to vehicle‐treated controls (Figure 8D). Doxorubicin also caused a pronounced reduction in total astrocyte number (Figure 8E), suggesting an inhibitory effect on cell survival or proliferation. Consistently, BrdU incorporation during a 24 h pulse was significantly lower in doxorubicin‐treated cultures than in controls (Figure 8F), indicating diminished DNA synthesis and cell proliferation. Despite these pronounced effects, there was no significant difference in LDH release between doxorubicin‐treated and control cells (Figure 8G), suggesting that doxorubicin did not induce substantial membrane damage or acute cytotoxicity under these conditions.

**FIGURE 9:**
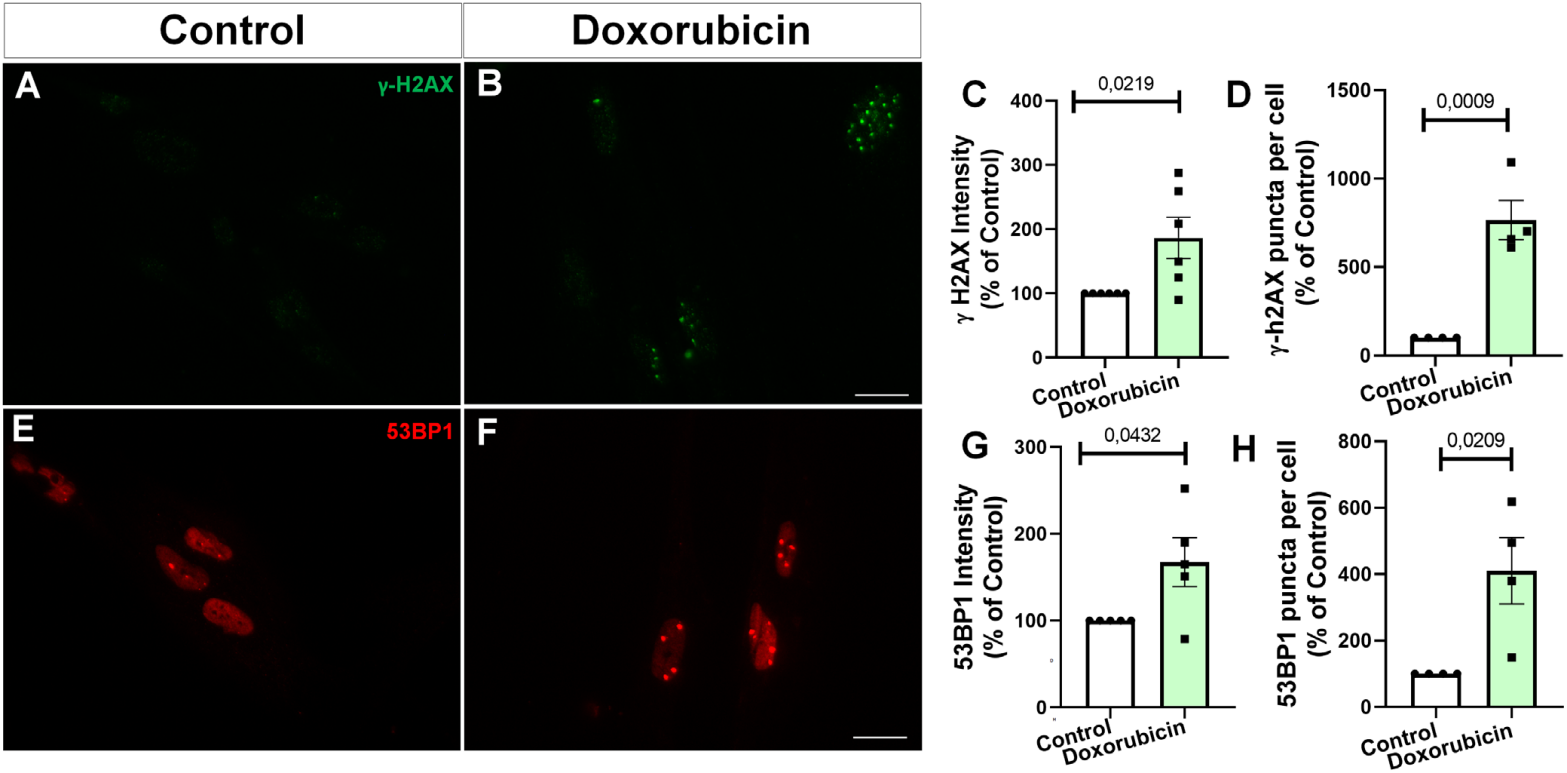
Doxorubicin induces a DNA damage response in primary human astrocytes. (A, B) Immunofluorescence images showing γ‐H2AX foci in control and doxorubicin‐treated astrocytes. (C) Quantification revealed increased γ‐H2AX intensity in the doxorubicin group (*t*(10) = 2.712, *p* = 0.0219). (D) The number of γ‐H2AX puncta per nucleus was markedly elevated (*t*(6) = 6.020, *p* = 0.0009). (E, F) Representative images of 53BP1 staining. (G) Quantification showed higher 53BP1 intensity in doxorubicin‐treated cells (*t*(8) = 2.400, *p* = 0.0432). (H) The number of 53BP1 puncta per cell was also increased (*t*(6) = 3.106, *p* = 0.0209). Bars represent mean ± SEM. Individual data points represent individual cultures (*n* = 4–6 per group). Unpaired two‐tailed Student's *t*‐test was used. Scale bars, 20 μm.

Supporting these findings, we found that doxorubicin‐treated primary human astrocytes showed activation of the DNA damage response, as demonstrated by increased intensity and distribution of nuclear puncta for γH2AX (Figures 9A–D) and 53BP1 (Figure 9E–H). Together, these results strongly indicate that doxorubicin treatment effectively induces a senescent phenotype in primary human astrocyte cultures.

## 8 Doxorubicin Induces a SASP in Human Astrocytes

**FIGURE 10:**
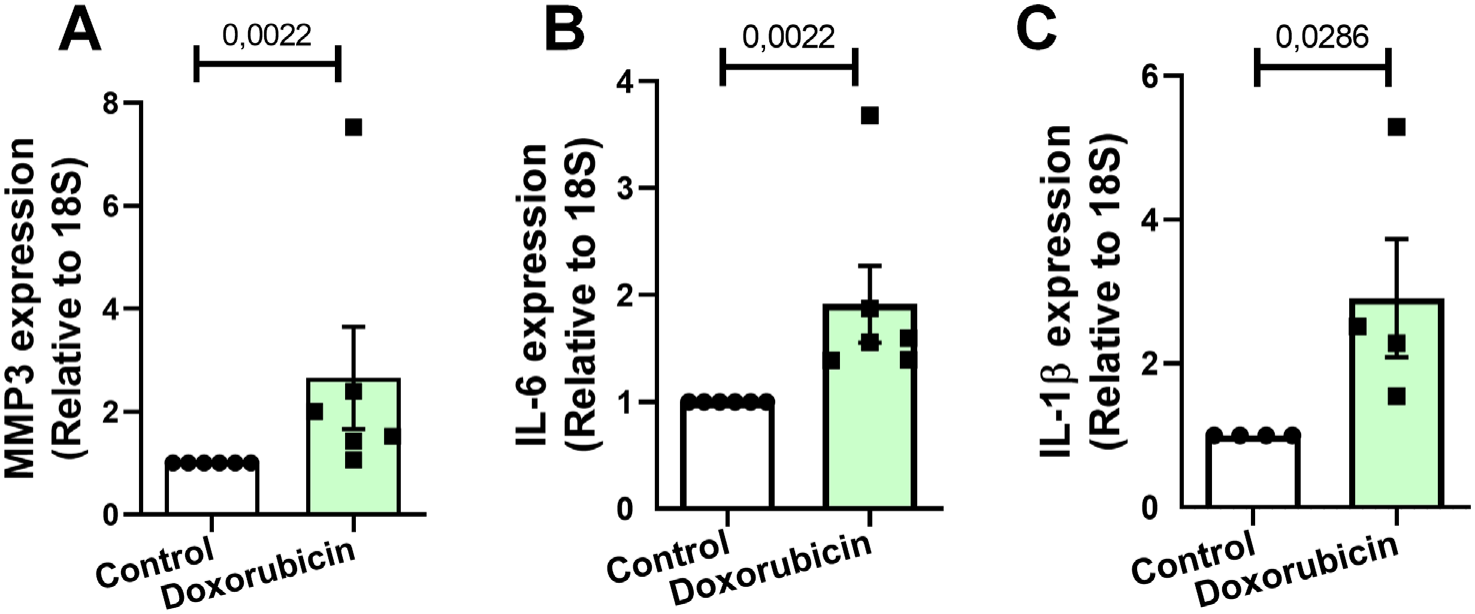
Doxorubicin induces a SASP phenotype in human astrocyte cultures. (A–C) mRNA expression of SASP‐associated genes was evaluated by qPCR in control and doxorubicin‐treated astrocytes using 18S as the endogenous reference gene. (A) MMP3 expression was significantly increased (*U* = 0, *p* = 0.0022). (B) IL‐6 mRNA levels were also upregulated (*U* = 0, *p* = 0.0022). (C) IL‐1β expression was elevated in doxorubicin‐treated cells (*U* = 0, *p* = 0.0286). Bars represent mean ± SEM. Individual data points represent individual cultures (*n* = 4–6 per group). Statistical analysis was performed using an unpaired two‐tailed Mann–Whitney *U* test. 18S: 18S ribosomal RNA.

To investigate whether doxorubicin induces the SASP phenotype in human astrocytes, we assessed the expression of key SASP markers. The results demonstrated that doxorubicin treatment significantly increased the expression of several critical markers in human astrocytes. Specifically, there was a marked upregulation of MMP3 expression (Figure 10A), a substantial rise in IL‐6 expression (Figure 10B), and elevated expression of IL‐1β (Figure 10C). These findings strongly indicate that doxorubicin induces a Senescence‐Associated Secretory Phenotype in human astrocytes, characterized by enhanced inflammatory signaling and matrix remodeling activity.

## 9 Discussion

In this study, we evaluated several chemical agents, including rotenone, lactacystin, and H_2_O_2_, for their potential to induce a senescent phenotype in primary murine astrocytes. Our results demonstrate that these agents were inconsistent in recapitulating the classical markers of cellular senescence, such as β‐galactosidase activity, phospho‐H2A.X, 53BP1 (DNA damage response marker), lamin B1, and p21 (Sivasubramanian et al. 2024; Bylicky et al. 2019; Bitto et al. 2010). In contrast, doxorubicin, a topoisomerase II inhibitor, successfully recapitulated the senescence‐associated phenotype, aligning with the established literature on its efficacy as a senescence inducer (Matias et al. 2022; Matias et al. 2019; Diniz et al. 2024).

In recent years, various experimental models and protocols have been employed to induce astrocytic senescence, including irradiation (Limbad et al. 2020), hydrogen peroxide (Gao et al. 2021; Simmnacher et al. 2020), rotenone (Simmnacher et al. 2020), etoposide (Bang et al. 2019), long‐term culturing (Bang et al. 2023), or a combination of AraC and long‐term culturing, as previously described by our group (Matias et al. 2022). These models effectively recapitulate several aspects of cellular senescence and the SASP phenotype; however, they each bear limitations, such as requiring prolonged in vitro culturing, inducing off‐target cellular damage, failing to fully mimic in vivo senescence dynamics, and sometimes triggering non‐specific stress responses that complicate the interpretation of results.

Rotenone is widely used as a mitochondrial toxin to model Parkinson's disease and other neurodegenerative conditions due to its ability to inhibit complex I of the electron transport chain and induce oxidative stress (Xiong et al. 2012). Despite its known role in triggering mitochondrial dysfunction and ROS production, rotenone did not consistently increase senescence markers such as β‐galactosidase activity and p21 expression in our primary mouse astrocyte cultures. These findings contrast with studies conducted in other cell types, where rotenone has been shown to activate senescence pathways through ROS‐mediated DNA damage and mitochondrial dysfunction (Simmnacher et al. 2020). The lack of response in astrocytes could be attributed to differences in exposure time and the concentration of rotenone, as it has been observed that rotenone induces senescence at nanomolar concentrations (Maurya et al. 2016) or micromolar concentrations (da Cruz et al. 2023), depending on the cell type.

Lactacystin, a potent proteasome inhibitor, has been employed in senescence studies to impair protein degradation and disrupt cellular homeostasis (Bitto et al. 2010). Previous research has indicated that proteasome inhibition can lead to a transient cell cycle arrest rather than a stable senescent state, especially in non‐transformed primary cells like astrocytes (Gavilan et al. 2015; Shenkman et al. 2007). However, similar to rotenone, lactacystin did not reliably induce senescence in our astrocyte cultures. The absence of significant alterations in markers such as lamin B1 and phospho‐H2A.X suggests that astrocytes may require a more prolonged exposure or higher concentration of lactacystin to activate the senescence program.

H_2_O_2_ is another widely used agent for inducing oxidative stress and senescence in vitro. Acute H_2_O_2_ exposure has been shown to cause DNA damage and activate the p53/p21 pathway, leading to cell cycle arrest (Driessens et al. 2009; Bitto et al. 2010). In our study, H_2_O_2_ treatment resulted in a mild increase in phospho‐H2A.X levels, indicating initial DNA damage; however, this was not sufficient to induce a sustained senescent phenotype. These results suggest that astrocytes possess a robust DNA repair capability that may counteract the senescence‐inducing effects of H_2_O_2_, or that the transient nature of the oxidative insult was insufficient to trigger irreversible senescence (Bylicky et al. 2019). Overall, our findings indicate that rotenone, lactacystin, and H_2_O_2_ are suboptimal for inducing a stable and reproducible senescence phenotype in primary murine astrocytes. The inconsistency observed with these agents highlights the need for further optimization of concentration, treatment duration, and combination strategies to achieve reliable senescence induction in these cells.

Doxorubicin is a potent chemotherapy drug commonly used to treat various cancers, including breast cancer, leukemia, and lymphoma. It belongs to the class of anthracyclines and functions by intercalating DNA strands, thereby inhibiting DNA and RNA synthesis. Doxorubicin induces double‐strand breaks (DSBs) in DNA, triggering cellular senescence as a result of DNA damage (Bielak‐Zmijewska et al. 2014; Oda et al. 2023). Additionally, doxorubicin generates ROS that induce oxidative stress, which can lead to DNA damage and trigger cellular senescence pathways (Kirsch et al. 2022). The accumulation of DNA disruption activates the DDR pathway, resulting in the activation of tumor suppressor proteins such as p53 and the upregulation of cyclin‐dependent kinase inhibitors like p21 (Jackson and Pereira‐Smith 2006). These events ultimately halt cell cycle progression, leading to cell cycle arrest and the acquisition of the senescent phenotype. Thus, doxorubicin's ability to induce DNA damage is a critical factor in its capacity to promote cellular senescence and its effectiveness as a chemotherapy agent (Sun et al. 2013).

Our data demonstrate that doxorubicin is a potent inducer of the senescent phenotype in astrocytes. The cells exhibited increased β‐galactosidase activity, elevated p21 levels, reduced lamin B1 levels, and an increase in DNA damage markers. Additionally, we observed an increase in the expression of SASP phenotype markers, including MMP3, IL‐6, and IL‐1β. Senescent cells exhibit distinct characteristics that differentiate them from healthy cells. A key marker is their ability to produce an enzyme called β‐galactosidase at a specific acidic pH. Additionally, senescent cells undergo cell cycle arrest mediated by the p21 protein interaction with CDK2, effectively halting cell division (Hernandez‐Segura et al. 2018). These changes are often accompanied by a decline in lamin B1, a protein crucial for maintaining proper nuclear structure (Matias et al. 2019; Matias et al. 2022).

Furthermore, senescent cells exhibit a unique secretory profile known as the SASP. This SASP encompasses a variety of molecules released by senescent cells, including growth factors, cytokines, and proteases. These secreted factors can have both positive and negative effects on surrounding cells (Ungerleider et al. 2021). Finally, DNA damage plays a significant role in triggering senescence. When DNA damage occurs, a rapid cellular response takes place, including the phosphorylation of a protein called H2A.X. This modified form, γ‐H2A.X, facilitates the study of DNA damage response pathways linked to senescence (Sharma et al. 2012). Another essential protein in DNA repair is 53BP1, which plays a crucial role in preserving genomic stability and coordinating the cellular response to stressors that may trigger senescence (Oda et al. 2023). Our findings corroborate its well‐established role, supporting the notion that 53BP1 recruitment to sites of DNA damage is dependent on the presence of γ‐H2A.X (Wang et al. 2002).

In this study, we employed an acute 24‐h treatment with doxorubicin, followed by a 14‐day recovery period in a low‐oxygen environment. Under these conditions, astrocytes exhibited hallmark features of senescence, including increased senescence‐associated SA‐β‐Gal activity, the presence of γH2AX foci, and upregulation of phosphorylated p53. These findings indicate that doxorubicin‐induced senescence in astrocytes can be effectively established under both physiological normoxic and hyperoxic culture conditions. However, further investigation is needed to delineate the functional and molecular differences elicited by these distinct oxygen environments. There is evidence indicating that doxorubicin can induce premature senescence in cardiomyocytes as well as in cardiac progenitor cells (Maejima et al. 2008). Doxorubicin induces senescence in microglia, leading to changes in their proteomes, increased cytokine secretion, and reduced capacity to internalize β‐amyloid (Marques et al. 2020).

Human astrocyte present distinct morphological and functional advantages over murine astrocyte, which are particularly significant for translational neuroscience research. Human astrocytes are notably larger, exhibit more complex branching structures, and display distinct calcium signaling profiles compared to their murine counterparts, which are simpler in form and function (Oberheim et al. 2009; Degl'Innocenti and Dell'Anno 2023). Genes related to defense response and metabolism differ between species, with human astrocytes showing greater susceptibility to oxidative stress than mouse astrocytes, largely due to variations in mitochondrial function and detoxification pathways (Li et al. 2021). These differences underscore the importance of human‐specific studies, especially in investigating diseases where astrocytes play key roles, such as neurodegenerative disorders. Additionally, astrocyte senescence models have shown that human astrocytes exhibit senescence features that are not fully replicated in murine models, including changes in gene expression linked to aging and inflammatory responses (Bhat et al. 2012). Building on previous studies, we developed a doxorubicin‐induced human astrocyte senescence model that effectively replicates key phenotypes characteristic of cellular senescence (Bitto et al. 2010; Crowe et al. 2016; Limbad et al. 2020; Simmnacher et al. 2020; Kim et al. 2024). Our model not only demonstrates hallmark features, such as β‐galactosidase activity, DNA damage markers, and the SASP, but also induces these phenotypes in a shorter culture time, reducing both cost and time. This approach provides a robust and efficient framework for studying senescence‐related astrocyte dysfunctions in a more human‐relevant setting.

In summary, this study highlights the challenges of inducing a consistent senescent phenotype in primary astrocytes using common chemical agents like rotenone, lactacystin, and H_2_O_2_. These agents failed to replicate the classical senescence markers observed with doxorubicin treatment, suggesting that alternative approaches or combinations may be necessary to achieve stable senescence induction in astrocytes. Doxorubicin proved to be a robust inducer of astrocyte senescence, altering all classical markers and promoting a pronounced SASP. The establishment of reliable astrocyte senescence models is essential for advancing our understanding of neurodegenerative processes and developing targeted interventions to alleviate the impact of cellular senescence in the CNS. Future studies should focus on optimizing senescence induction protocols and exploring the therapeutic potential of modulating astrocyte senescence in neurodegenerative disease models.

## Author Contributions

**Mariana Marques:** validation, visualization, investigation, formal analysis, data curation. **Lívia de Sá Hayashide:** validation, investigation, formal analysis. **Pedro Amorim:** investigation, validation, formal analysis. **Beatriz Martins Fernandes:** investigation, validation, formal analysis, visualization. **Ana Paula Bergamo Araujo:** investigation, formal analysis, validation. **Daniel Fernandes Messor:** investigation, validation, formal analysis. **Vitor Emanuel Leocadio:** investigation, validation, formal analysis. **Bruna Pessoa:** investigation. **João Bastos Lima Pacca Corrêa:** investigation, validation, formal analysis. **Cristopher Villablanca:** investigation, validation, formal analysis, writing – review and editing. **René L. Vidal:** investigation, validation, formal analysis. **Christian González‐Billault:** writing – review and editing, supervision, funding acquisition, resources. **Isadora Matias:** supervision, writing – review and editing, investigation, formal analysis, validation, visualization. **Flávia Carvalho Alcantara Gomes:** writing – review and editing, formal analysis, supervision, funding acquisition, resources. **Luan Pereira Diniz:** conceptualization, investigation, validation, formal analysis, supervision, project administration, writing – original draft, writing – review and editing, visualization, funding acquisition, methodology, resources.

## Consent

Informed consent was achieved for all subjects, and the experiments were approved by the local ethics committee.

## Conflicts of Interest

The authors declare that they have no financial or non‐financial conflicts of interest related to the work reported in this manuscript. Christian Gonzalez‐Billault is Senior Editor of the Journal of Neurochemistry. This role had no influence on the editorial process or review of this manuscript.

## Peer Review

The peer review history for this article is available at https://www.webofscience.com/api/gateway/wos/peer‐review/10.1111/jnc.70177.

## Supporting information


**Data S1:** jnc70177‐sup‐0001‐supinfo.pdf.

## Data Availability

The data that support the findings of this study are available from the corresponding author upon reasonable request.
