# Supplementary material for: Doxorubicin Induces a Senescent Phenotype in Murine and Human Astrocytes
Source: J Neurochem. 2025 Aug 5;169(8):e70177. doi: 10.1111/jnc.70177 (PMC12322803; doi:10.1111/jnc.70177)
Supplement: Supplementary file 1 — Data S1: jnc70177‐sup‐0001‐supinfo.pdf. [file JNC-169-0-s001.pdf]

SUPPORTING INFORMATION

Doxorubicin induces a senescent phenotype in murine and human astrocytes.

Mariana Marques[1+], Lívia de Sá Hayashide[1+], Pedro Amorim[1], Beatriz Martins Fernandes[1], Ana Paula Bergamo Araujo[1], Daniel Fernandes Messor[1], Vitor Emanuel Leocadio[1], Bruna Pessoa[1], João Bastos Lima Pacca Corrêa[1], Cristopher Villablanca [2,4], René Vidal [3], Christian González-Billault [2,4,5], Isadora Matias[1], Flávia Carvalho Alcantara Gomes[1] and Luan Pereira Diniz[1*]

[1] Instituto de Ciências Biomédicas, Universidade Federal do Rio de Janeiro, Rio de Janeiro, Brasil.
2 Cell and Neuronal Dynamics Laboratory, Department of Biology, Faculty of Sciences, Universidad de Chile, Santiago, Chile
3 Center for Integrative Biology, Universidad Mayor, Santiago, Chile
4 Geroscience Center for Brain Health and Metabolism (GERO), Santiago, Chile
5 The Buck Institute for Research on Aging, Novato, USA

[+] These authors contributed equally to this work

*Corresponding author:

SUPPLEMENTARY MATERIAL AND METHODS

Western-Blotting

Astrocytes were lysed using RIPA buffer supplemented with protease inhibitors (Complete™ Mini, Roche), collected via cell scrapers, and centrifuged to obtain cleared lysates (14,000 rpm, 4ºC, 20 min). Protein concentration was determined using a Qubit Protein BR Assay Kit (ThermoFisher). A total of 20 µg of protein samples in loading buffer were heat-denatured at 98ºC for 5 minutes and subsequently separated on TGX Stain-Free™ FastCast™ gels (Bio-Rad). Total protein was visualized through UV transillumination for 1–5 minutes and imaged utilizing a Chemidoc imaging system (Uvitec). Subsequently, proteins were transferred onto PVDF membranes (Immobilon-FL, Millipore) using a Power Blotter–Semi-dry Transfer System (ThermoFisher). Membranes were blocked with 5% BSA in Tris-Buffered Saline containing 0.1% Tween-20 (TBS-T) for 1 hour and incubated overnight at 4ºC with the following primary antibodies prepared in TBS-T with 1% BSA: Lamin-B1 (1:1000, ab229025, Abcam, RRID:AB_3083735), phospho-p53 (1:1000, #9284, Cell Signaling Technology, RRID:AB_331464), and β-actin (C4) (1:1000, sc-47778, Santa Cruz Biotechnology, RRID:AB_626632). After washing with TBS-T (three times for 5 minutes each), membranes were incubated for 1 hour at room temperature with species-appropriate HRP-conjugated secondary antibodies (715-035-150, RRID: AB_2340770 and 711-035-152, RRID: AB_10015282; Jackson ImmunoResearch) and then washed again with TBS-T (three times for 5 minutes each). Signal detection was performed using SuperSignal™ West Dura chemiluminescent substrate (Thermo Scientific), and images were acquired using a Chemidoc system (Uvitec). Stain-Free total protein normalization was achieved by UV transillumination for 1–5 minutes, and the corresponding image was captured using the same imaging system. Band intensities were quantified via densitometric analysis in ImageJ.

Characterization of murine and human astrocyte cultures

For immunocytochemical characterization, murine and human astrocyte cultures were fixed and processed according to the same protocol described in the Experimental Procedures section of the manuscript, with the only difference being the dilution of the primary antibodies. After fixation, cells were incubated in blocking solution (composition as described previously) to prevent nonspecific binding, followed by overnight incubation at 4 °C with the following primary antibodies: rabbit anti-GFAP (1;1,000; DakoCytomation, Glostrup, Denmark, RRID:AB_10013382), mouse anti-S100β (1:1,000 dilution, Sigma-Aldrich, Cat. S2532, RRID:AB_477499), and rat anti-F4/80 (Bio-Rad Cat. MCA497G, RRID:AB_872005). After thorough washing, cells were incubated with species-specific Alexa Fluor-conjugated secondary antibodies (1:1000; Invitrogen) for 2 hours at room temperature. Nuclei were counterstained with Hoechst 33342, and coverslips were mounted using aqueous mounting medium (Sigma-Aldrich). Images were acquired using an epifluorescence microscope under identical acquisition settings across all experimental groups. Quantification was performed by calculating the percentage of marker-positive cells relative to the total number of Hoechst-stained nuclei.

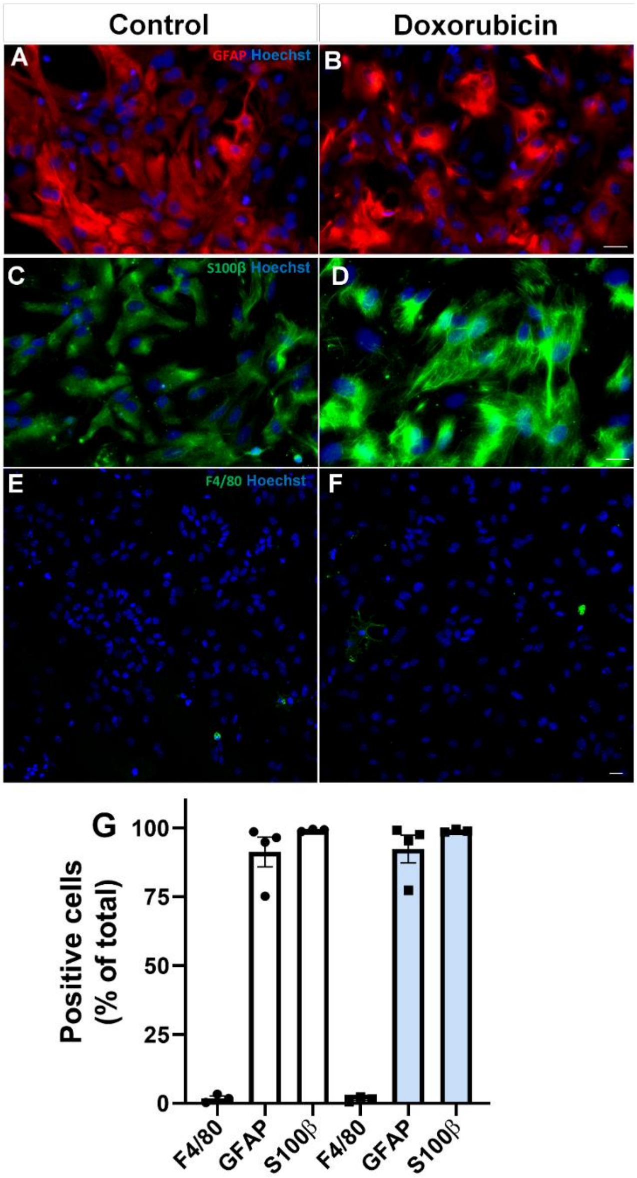

Supplementary Figure 1. Doxorubicin-treated cultures remain highly enriched in astrocytes. Immunocytochemistry was performed to evaluate cell-type identity in control and doxorubicin-treated cultures. (A–B) Astrocytes were positive for GFAP (red) and (C–D) S100β (green), with nuclei counterstained using Hoechst (blue). (E–F) Immunostaining for F4/80 (green), a microglial marker, revealed minimal contamination in both groups. (G) Quantification confirmed that over 95% of cells were $GFAP^{+}$ and $S100\beta^{+}$, while less than 3% were $F4/80^{+}$, with no significant differences between control and doxorubicin-treated cultures. Bars represent mean ± SEM (n = 3-4 independent cultures). Scale bars = 20 µm.

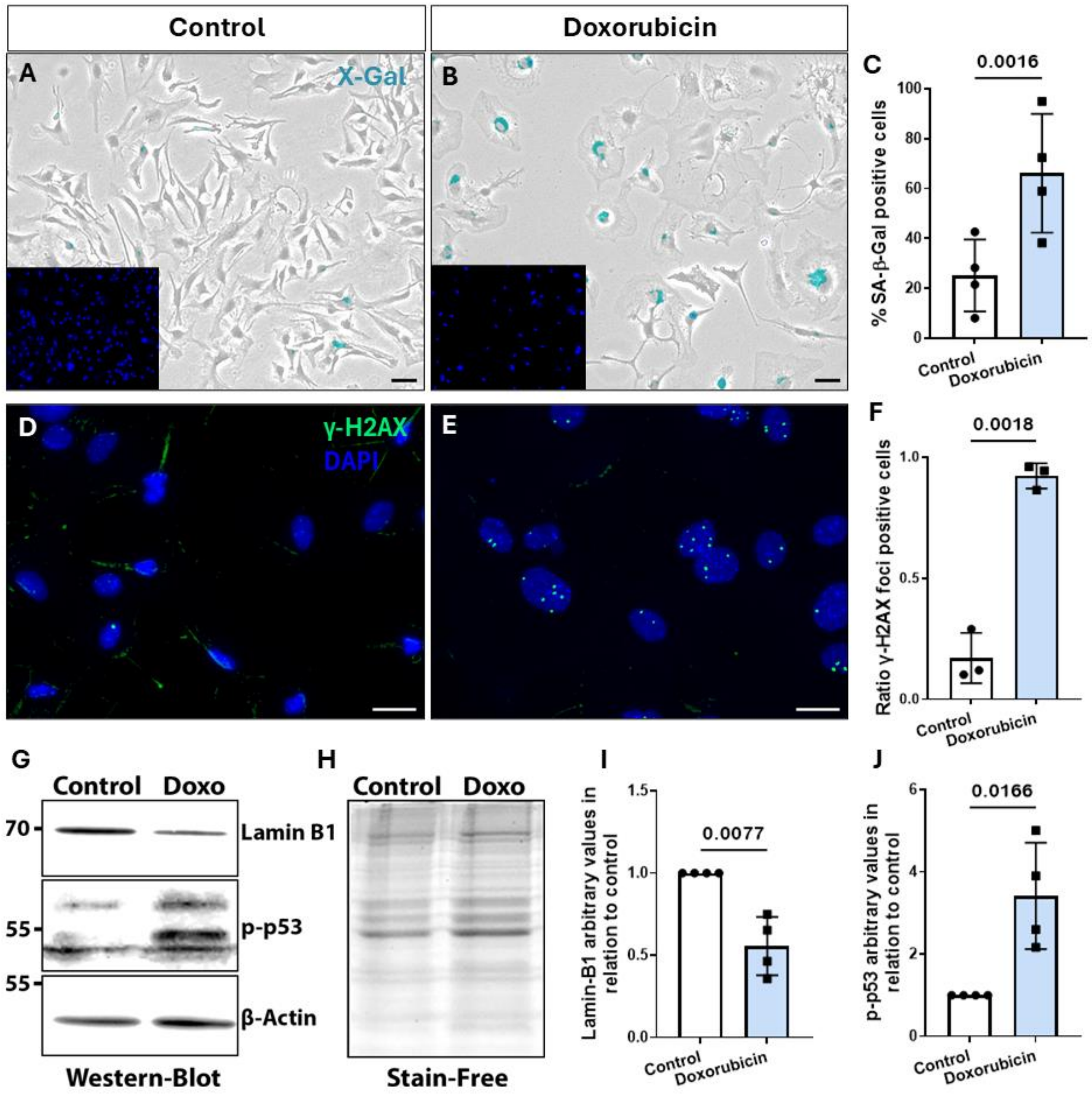

Supplementary Figure 2. Doxorubicin induces long-term senescence in murine astrocytes. Primary astrocyte cultures were treated with 250 nM doxorubicin for 24 h and maintained under low-oxygen conditions (5% CO_2, 3.5% O_2) for 14 days. Control cells were cultured for 2 days. Under these conditions, doxorubicin-treated astrocytes exhibited increased SA-β-galactosidase activity (A–C; $t(3) = 8.681$, $p = 0.0016$) and elevated nuclear γ-H2AX foci (D–F; $t(2) = 16.74$, $p = 0.0018$). Western blot analysis normalized to total protein (Stain-Free) showed decreased Lamin B1 expression (G–I; $t(3) = 5.010$, $p = 0.007$) and increased phosphorylated p53 (G, H, J; $t(3) = 3.744$, $p = 0.0166$), relative to controls. Statistical analysis was performed using one-tailed paired Student's *t*-test. Error bars represent mean ± SD. Scale bars: 20 μm.

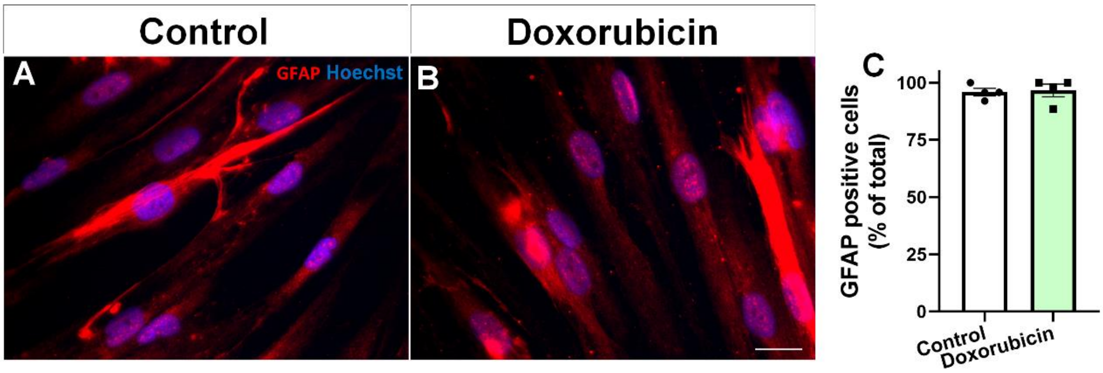

Supplementary Figure 3. Doxorubicin treatment does not alter astrocyte identity. (A–B) Representative images of GFAP (red) and Hoechst (blue) staining in control and doxorubicin-treated astrocytes. (C) Quantification shows that the proportion of GFAP-positive cells remained above 95% in both groups, indicating preserved astrocytic identity. Bars represent mean ± SEM (n = 4 independent cultures). Scale bar = 20 μm.

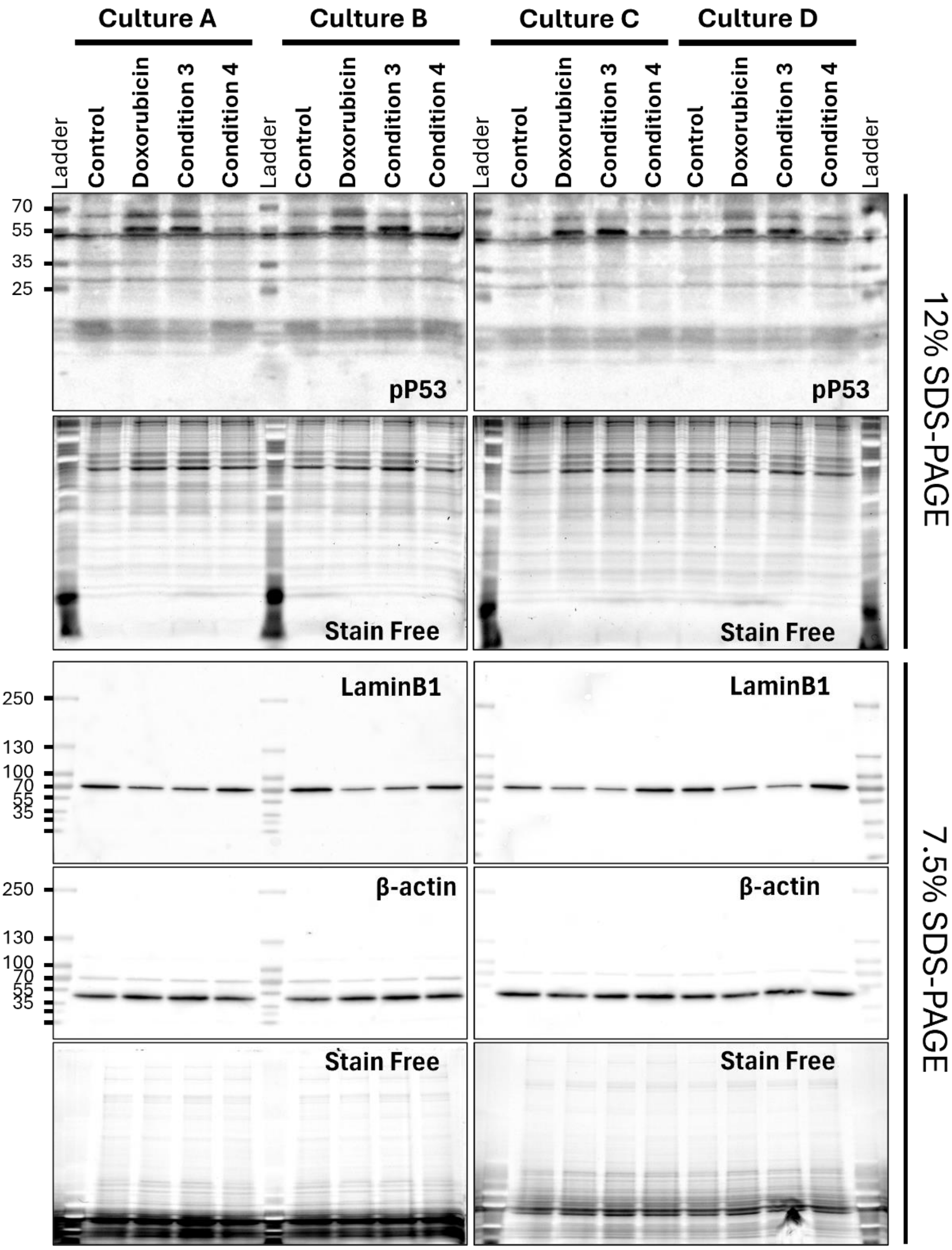

Supplementary Figure 4. Full Western blot membranes. A total of 20 µg of protein from four independent astrocyte cultures was resolved on 12% or 7.5% SDS-PAGE gels, as indicated. Lanes corresponding to control and doxorubicin-treated conditions are labeled; conditions 3 and 4 (exploratory treatments) are not included in the main analysis. Stain-Free imaging was performed according to the manufacturer's protocol and demonstrates comparable total protein loading across lanes. β-actin levels remained stable across conditions and were not significantly altered by treatment.
